# Cultivar and Soil Properties Jointly Shape the Rhizosphere Microbial Communities and Biochemical Properties Associated with Ginger (*Zingiber officinale*) Productivity

**DOI:** 10.3390/ijms27188428

**Published:** 2026-09-21

**Authors:** Panpan Yu, Liu Huang, Xinju Wang, Shu Han, Songmei Shi

**Affiliations:** Key Laboratory of Vegetable Biology in Yunnan, College of Landscape and Horticulture, Yunnan Agricultural University, Kunming 650201, China

**Keywords:** *Zingiber officinale*, rhizosphere microorganism, cultivar variation, soil enzyme activity, soil nutrient availability, ginger yield

## Abstract

Whether high-yielding cultivars recruit a universally beneficial microbial community or deploy location-specific strategies remains unknown. To address this, we evaluated two ginger cultivars (‘Dianjiang G04’ and ‘Dianjiang G07’) grown at two locations with contrasting soil physicochemical properties, Kunming (KM) and Luoping (LP), Yunnan Province, China. Ginger yield, soil physicochemical properties, enzyme activities, and rhizosphere bacterial and fungal communities were investigated using a two-site × two-cultivar factorial design. Cultivar exerted the strongest effect on yield, with G07 outyielding G04 and KM-G07 producing the highest yield. Compared with Luoping, Kunming soils generally exhibited higher pH, NO_3_^−^-N, available phosphorus, available potassium, enzyme activities, and alpha diversity. Bacterial and fungal communities were clearly differentiated between locations. Additionally, cultivar-related community divergence was more pronounced in Luoping than in Kunming. Redundancy analysis showed that pH, total phosphorus, total potassium, invertase, urease, and protease were closely associated with microbial variation, while functional predictions revealed location- and cultivar-dependent shifts in bacterial metabolism and fungal trophic guilds. Partial least squares regression identified cultivar, nutrients, enzyme activities, *Trichocladium*, and *Bacillus* as key yield predictors. These findings indicate that ginger performance depends on cultivar–soil properties compatibility rather than on cultivar selection alone: cultivar determines yield, but soil properties shape the microbial community, confirming that high-yielding cultivars achieve superiority through site-specific microbial recruitment. Therefore, G07 should be prioritized in soils with more favorable pH conditions and greater N, P, and K availability, whereas in less fertile or acidic soils, alternative cultivars or targeted soil amelioration may be more appropriate.

## 1. Introduction

Ginger (*Zingiber officinale* Roscoe) is an economically important spice and medicinal crop widely cultivated in tropical and subtropical regions. Its rhizome is valued not only as a food seasoning but also as a medicinal resource due to its diverse bioactive compounds. However, the stable production of ginger is increasingly challenged by yield instability and soil degradation under intensive cultivation systems. In many ginger-growing regions, repeated monoculture or continuous cropping is commonly practiced because of limited suitable land resources and high market demand. Although this planting pattern can maintain short-term production, long-term continuous cropping frequently induces a series of soil problems, including nutrient imbalance, soil acidification or salinization, deterioration of soil structure, accumulation of soil-borne pathogens, and disruption of rhizosphere microbial communities. These changes ultimately reduce crop productivity and threaten the sustainability of ginger production systems [1,2,3].

As a fundamental substrate for plant growth, soil health is essential for maintaining crop productivity and agroecosystem stability. It is jointly regulated by physicochemical properties, including soil structure, pH, organic matter, and nutrient availability, as well as biological processes, such as soil enzyme activities and microbial community functions, which collectively influence root development, nutrient cycling, disease suppression, and crop performance [1,4,5,6,7]. Among soil biological components, microorganisms are core drivers of biogeochemical cycles [6,8]. Bacteria promote nitrogen transformation through processes such as ammonification and nitrification, thereby enhancing nutrient availability [9]. Fungi, in turn, form extensive hyphal networks that improve soil structural stability and establish symbiotic relationships with plant roots, such as arbuscular mycorrhizal fungi, which assist host plants in acquiring mineral nutrients, including phosphorus and zinc [10,11,12]. The functional interactions between fungi and bacteria are important biological drivers that maintain soil fertility and promote crop growth [10,13]. Furthermore, soil enzyme activities are direct indicators of microbial function and serve as an important bridge linking microbial communities with soil nutrient cycling. For example, invertase participates in carbon metabolism, urease and protease are involved in nitrogen transformation, and phosphatase regulates phosphorus availability. Changes in these enzyme activities can sensitively reflect soil health status and nutrient supply capacity [7]. Accumulating evidence has linked rhizosphere microbial community composition to crop yield in various agricultural systems. For example, specific microbial taxa, such as plant growth-promoting rhizobacteria (PGPR) and mycorrhizal fungi, have been shown to enhance nutrient uptake, suppress soil-borne pathogens, and stimulate plant hormone signaling, ultimately contributing to increased yields [14,15,16]. Conversely, dysbiosis or shifts toward pathogenic microbial communities can lead to yield decline, as observed in continuous cropping systems of various crops [17,18]. Despite the recognized importance of rhizosphere microbial communities in crop performance, most existing studies on ginger have focused on single-location comparisons or pathogen-related shifts under controlled conditions. Whether and how cultivar genetic identity versus local soil properties shapes the rhizosphere microbial community composition—and its subsequent impact on yield—remain open questions under field conditions, particularly in ginger production systems. It is largely unknown whether a high-yielding cultivar maintains its superiority by recruiting a universally beneficial microbial community across different soil conditions or by deploying site-specific recruitment strategies.

To address this issue, we established a two-site × two-cultivar field experiment in Yunnan Province, China, using two widely grown ginger cultivars (‘Dianjiang G04’ and ‘Dianjiang G07’), selected from a preliminary multi-location screening trial for their contrasting yield performance (G04: lowest; G07: highest), at two distinct growing locations (Kunming and Luoping), which differ in naturally occurring soil physicochemical properties. This 2 × 2 factorial design allowed us to: (i) assess whether the rhizosphere microbial community of the same cultivar differs between the two locations with distinct soil properties (location-driven variation); (ii) determine whether dominant microbial taxa differ between the high-yielding (G07) and low-yielding (G04) cultivars within the same location (cultivar-driven variation); and (iii) quantify the relative contributions and interaction effects of cultivar, location, and their combination on ginger yield. We hypothesized that soil conditions would exert a stronger influence on microbial community assembly than cultivar but that specific cultivar-enriched taxa would serve as key predictors of yield performance, particularly where soil pH and nutrient availability differ markedly between locations. To test these hypotheses, we integrated physicochemical profiling, enzyme activity assays, amplicon sequencing (16S and ITS), and multivariate statistical modeling (RDA, Spearman correlation, and PLSR-VIP), supplemented with exploratory functional predictions based on the FUNGuild and KEGG databases. This integrative approach provides a comprehensive understanding of how cultivar and soil properties jointly shape rhizosphere microbial communities and ultimately determine ginger yield under field conditions.

## 2. Results

### 2.1. Ginger Yield and Soil Physicochemical Properties

**Figure 1:**
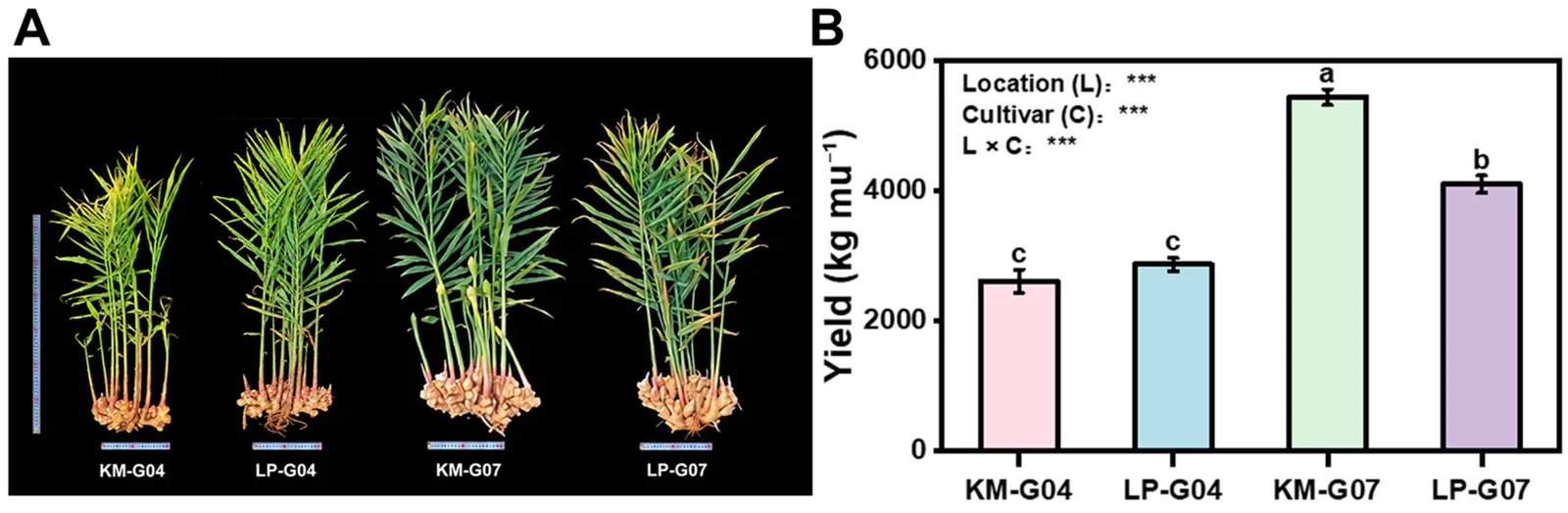
Plant morphology (**A**) and yield (**B**) of two ginger cultivars (G04 and G07) grown in Kunming (KM) and Luoping (LP). Data are shown as mean ± SE (*n* = 3). Different lowercase letters indicate significant differences among treatments (*p* < 0.05). Asterisks indicate significance levels: *** indicates statistical significance at *p* < 0.001.

In terms of crop yield, cultivar G07 consistently achieved much higher yields than G04 at both Kunming and Luoping. While the yield of cultivar G07 was significantly higher in Kunming than in Luoping, G04 yield did not differ significantly between the two locations (Figure 1).

Soil properties were differentially affected by location and cultivar. Location had significant effects on pH, TP, TK, NO_3_^−^-N, and AK (*p* < 0.05 or *p* < 0.01), whereas cultivar significantly influenced SOC and TN (*p* < 0.01). Specifically, soil pH in Kunming (7.71–8.00) was significantly higher than in Luoping (6.20–6.32). Similarly, Kunming soils exhibited 1.4–1.5-fold higher NO_3_^−^-N (55–69 mg kg^−1^ vs. 39–47 mg kg^−1^) and 1.3–1.4-fold higher AK (364–378 mg kg^−1^ vs. 272–274 mg kg^−1^) than Luoping soils. In contrast, TK and TP contents were significantly higher in Luoping soil. In terms of cultivar effects, G07 showed significantly higher TN and SOC contents than G04. A significant cultivar × location interaction was observed only for TP (*p* < 0.01) (Table 1).

### 2.2. Soil Enzyme Activities

**Figure 2:**
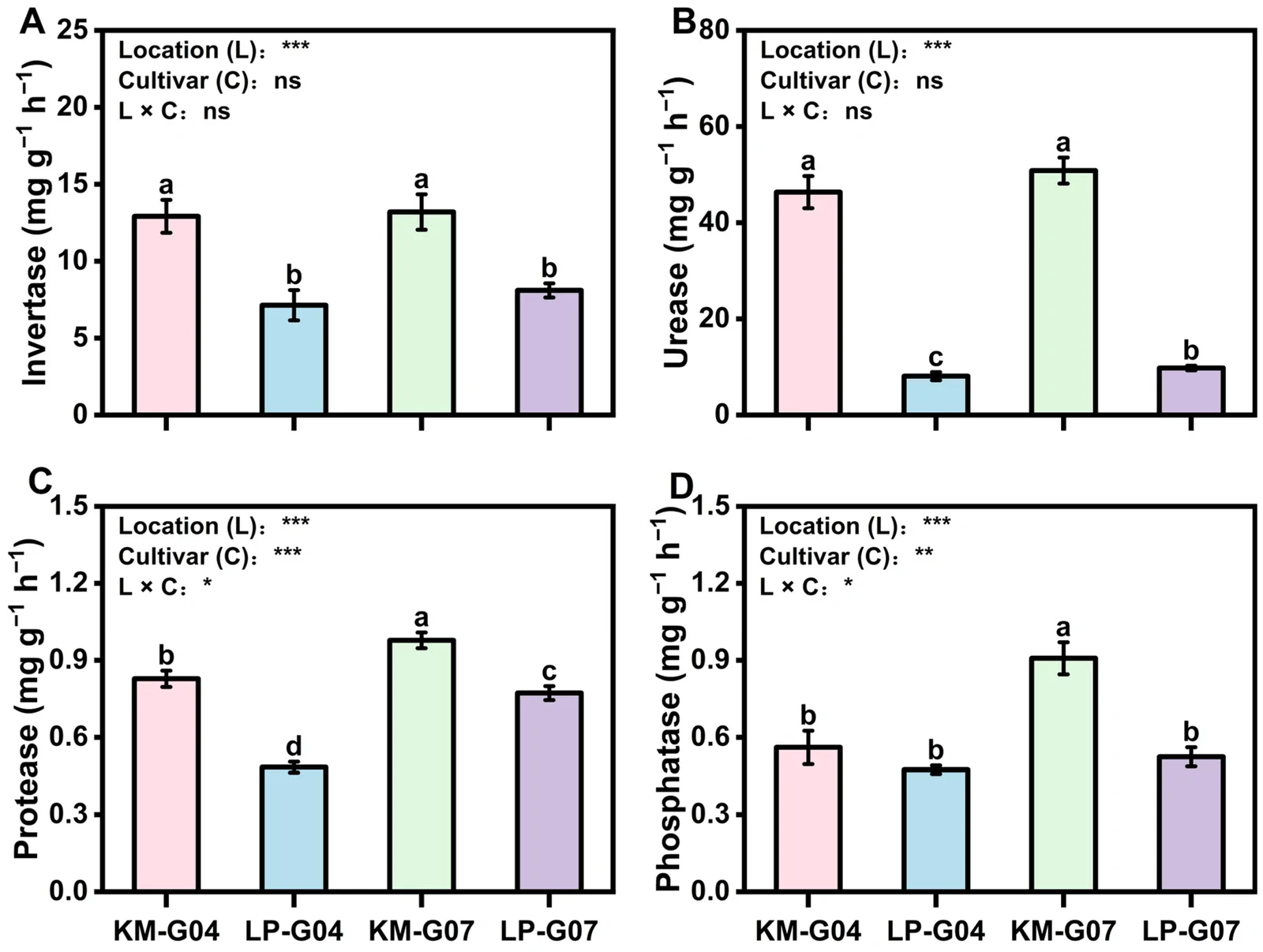
Soil enzyme activities ((**A**) invertase, (**B**) urease, (**C**) protease, and (**D**) phosphatase) under different treatments, consisting of two ginger cultivars (G04 and G07) grown at two locations (KM and LP). Data are shown as mean ± SE (*n* = 3). Different lowercase letters indicate significant differences among treatments (*p* < 0.05). Asterisks indicate significance levels: ns indicates no statistically significant difference, * *p* < 0.05, ** *p* < 0.01, and *** *p* < 0.001.

As shown in Figure 2A–D, the soils in Kunming exhibited consistently higher invertase, urease, and protease activities than those in Luoping. G07 showed higher urease activity in Luoping, higher phosphatase activity in Kunming, and higher protease activity in both Kunming and Luoping than G04. No significant difference in invertase activity was observed between G07 and G04 at either location.

### 2.3. Diversity of Rhizosphere Bacterial and Fungal Communities

**Figure 3:**
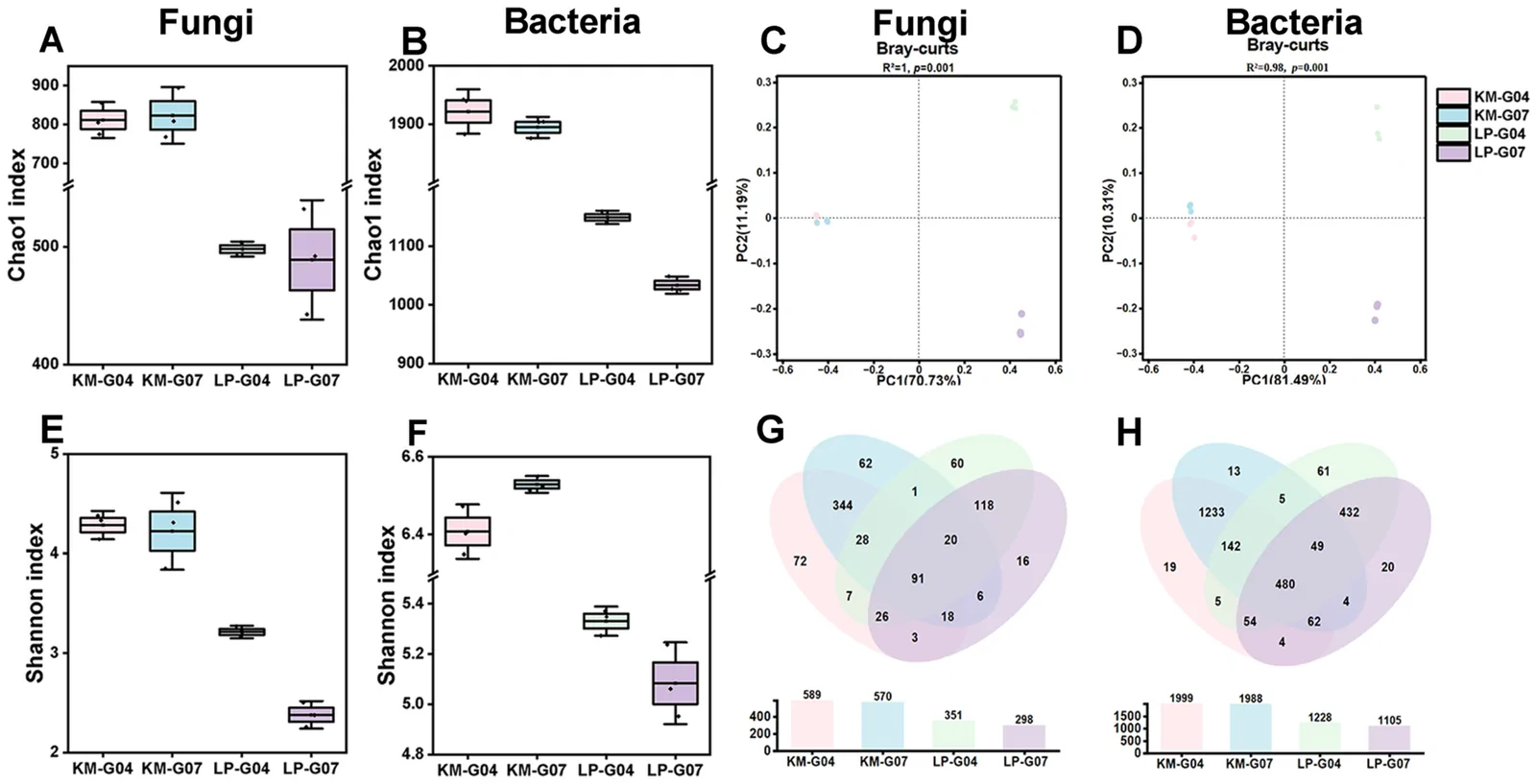
Diversity and community structure of rhizosphere bacterial and fungal communities under different location–cultivar combinations. (**A**–**D**) Alpha diversity indices: Chao1 richness (**A**,**B**) and Shannon diversity (**C**,**D**) for fungal and bacterial communities, respectively. (**E**,**F**) Principal coordinate analysis (PCoA) plots based on Bray–Curtis distances showing β-diversity of fungal (**E**) and bacterial (**F**) communities. (**G**,**H**) Venn diagrams illustrating shared and unique ASVs of fungi (**G**) and bacteria (**H**) across the four treatments. KM and LP indicate Kunming and Luoping, respectively; G04 and G07 represent the two cultivars. Data are shown as mean ± SE (*n* = 3).

Alpha diversity indices revealed distinct microbial community differences between the growing locations and ginger cultivars. For fungal communities, both Chao1 richness and Shannon diversity were higher in Kunming than in Luoping (Figure 3A,E). Chao1 richness did not differ significantly between the cultivars at either location, and Shannon diversity was higher in G04 than in G07 in Luoping. For bacterial communities, both the Chao1 richness index and Shannon diversity index were also higher in Kunming than in Luoping. G07 had a higher Shannon diversity index than G04 in Kunming soil, but G07 had a lower Chao1 richness and Shannon diversity than G04 in Luoping (Figure 3B,F). Overall, location exerted a stronger influence on alpha diversity than cultivar. Principal coordinate analysis (PCoA) revealed that the fungal and bacterial communities were distinctly separated between Kunming and Luoping (Figure 3C,D). Within each location, the two cultivars were also segregated, with a more pronounced separation observed in Luoping than in Kunming.

### 2.4. Taxonomic Composition of Bacterial and Fungal Communities

Venn diagram analysis revealed treatment-specific ASV richness and community structure. A core set of 91 fungal and 480 bacterial ASVs was shared among all four treatments. Notably, the number of shared ASVs between the cultivars was higher in Kunming than in Luoping for both bacteria (1233 vs. 432) and fungi (344 vs. 118) (Figure 3G,H).

**Figure 4:**
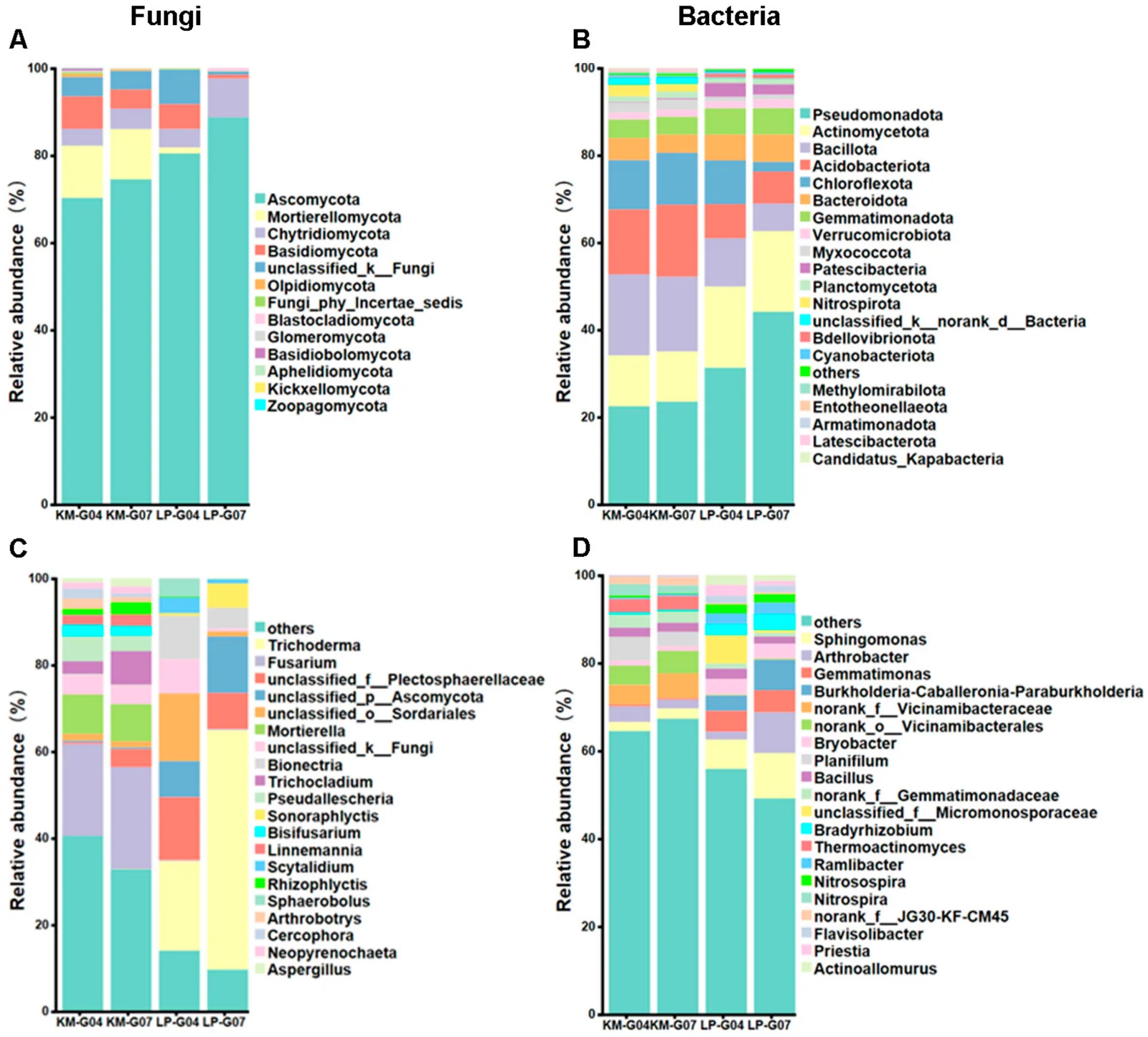
Comparison of fungal and bacterial communities in the rhizosphere of two ginger (*Zingiber officinale* Roscoe) cultivars, G04 and G07, grown at two locations, Kunming (KM) and Luoping (LP). (**A**,**B**) Relative abundance of dominant microbial phyla ((**A**) fungi; (**B**) bacteria). (**C**,**D**) Relative abundance of dominant microbial genera ((**C**) fungi; (**D**) bacteria). Taxa with low relative abundance were grouped as “others”.

At the phylum level, the rhizosphere fungal community was dominated by *Ascomycota*, with relative abundances ranging from 70.3% to 88.7% across the four treatments (Figure 4A). *Ascomycota* was more abundant in the Luoping treatments than in the Kunming treatments, particularly in LP-G07. Other major fungal groups included *Mortierellomycota*, *Chytridiomycota*, *Basidiomycota*, and *unclassified_k__Fungi*. *Mortierellomycota* was more abundant in KM-G04 and KM-G07 but occurred at markedly lower abundances in LP-G04 and LP-G07. For bacteria, the dominant phyla were *Pseudomonadota*, *Actinomycetota*, *Bacillota*, *Acidobacteriota*, and *Chloroflexota* (Figure 4B). *Pseudomonadota* and *Actinomycetota* were more abundant in LP-G04 and LP-G07 than in KM-G04 and KM-G07, whereas *Bacillota*, *Acidobacteriota*, and *Chloroflexota* were relatively more abundant in the Kunming treatments.

At the fungal genus level, community composition differed markedly among the four location–cultivar combinations (Figure 4C). *Fusarium* was abundant in KM-G04 and KM-G07, accounting for 21.3% and 23.3% of the fungal community, respectively, but occurred at very low abundances in LP-G04 and LP-G07. In contrast, *Trichoderma* was more abundant in the Luoping treatments, particularly in LP-G07, where it accounted for 55.3% of the total fungal abundance. *Mortierella* was also more abundant in the Kunming treatments than in the Luoping treatments. At the bacterial genus level, the major identified taxa included *Sphingomonas*, *Arthrobacter*, *Gemmatimonas*, *Planifilum*, *Bacillus*, and *norank_f__Gemmatimonadaceae* (Figure 4D).

### 2.5. LEfSe Analysis of Rhizosphere Fungi and Bacteria

**Figure 5:**
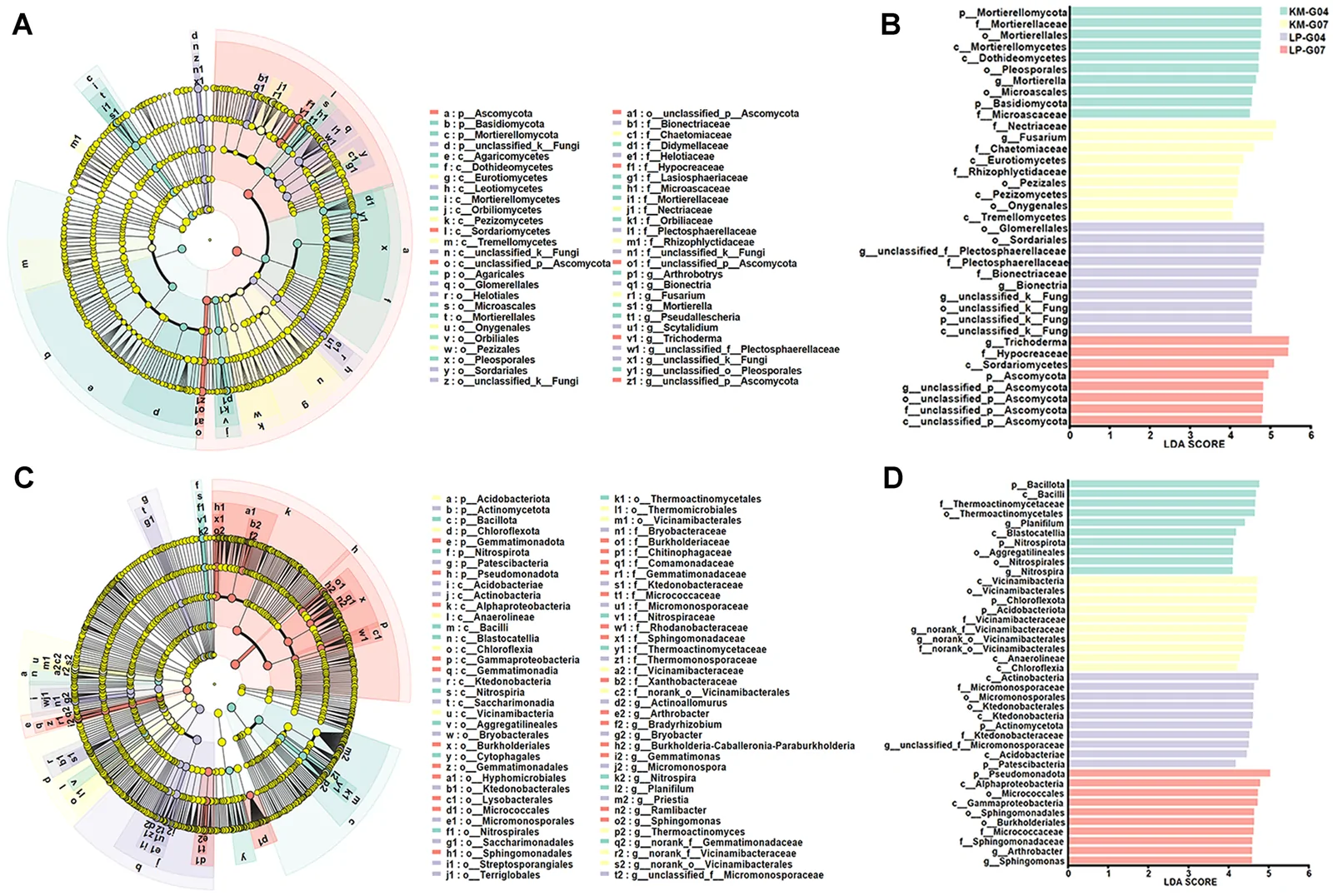
LEfSe analysis of fungal (**A**,**B**) and bacterial (**C**,**D**) taxa in ginger rhizosphere soil. Circles represent taxonomic levels from phylum to genus, with node size proportional to the relative abundance. Bar plots show the LDA scores for significantly different taxa, illustrating the differential impact of key biomarker taxa across treatments.

LEfSe analysis (LDA score > 4) identified treatment-enriched microbial taxa across the four location–cultivar combinations (Figure 5). For the fungal community, KM-G04 was predominantly enriched in Mortierellomycota (Mortierellomycetes → Mortierellales → Mortierellaceae). KM-G07 was mainly enriched in *Fusarium* and its affiliated family Nectriaceae, as well as Chaetomiaceae. LP-G04 was characterized by significant enrichment of Glomerellales, Sordariales, and unclassified_f_Plectosphaerellaceae. In contrast, LP-G07 was mainly enriched in Ascomycota, Sordariomycetes, Hypocreaceae, and *Trichoderma* (Figure 5A,B).

For the bacterial community, KM-G04 was predominantly enriched in Bacillota (Bacilli → Thermoactinomycetales → Thermoactinomycetaceae). KM-G07 was mainly enriched in Chloroflexota, Acidobacteriota, Vicinamibacteria, and Vicinamibacterales. LP-G04 was characterized by significant enrichment of Actinobacteria, Ktedonobacterales-related taxa, Micromonosporales, and Micromonosporaceae. In contrast, LP-G07 was mainly enriched in Pseudomonadota, Alphaproteobacteria, Gammaproteobacteria, and Micrococcales (Figure 5C,D).

### 2.6. Functional Predictions of Rhizosphere Bacterial and Fungal Communities

**Figure 6:**
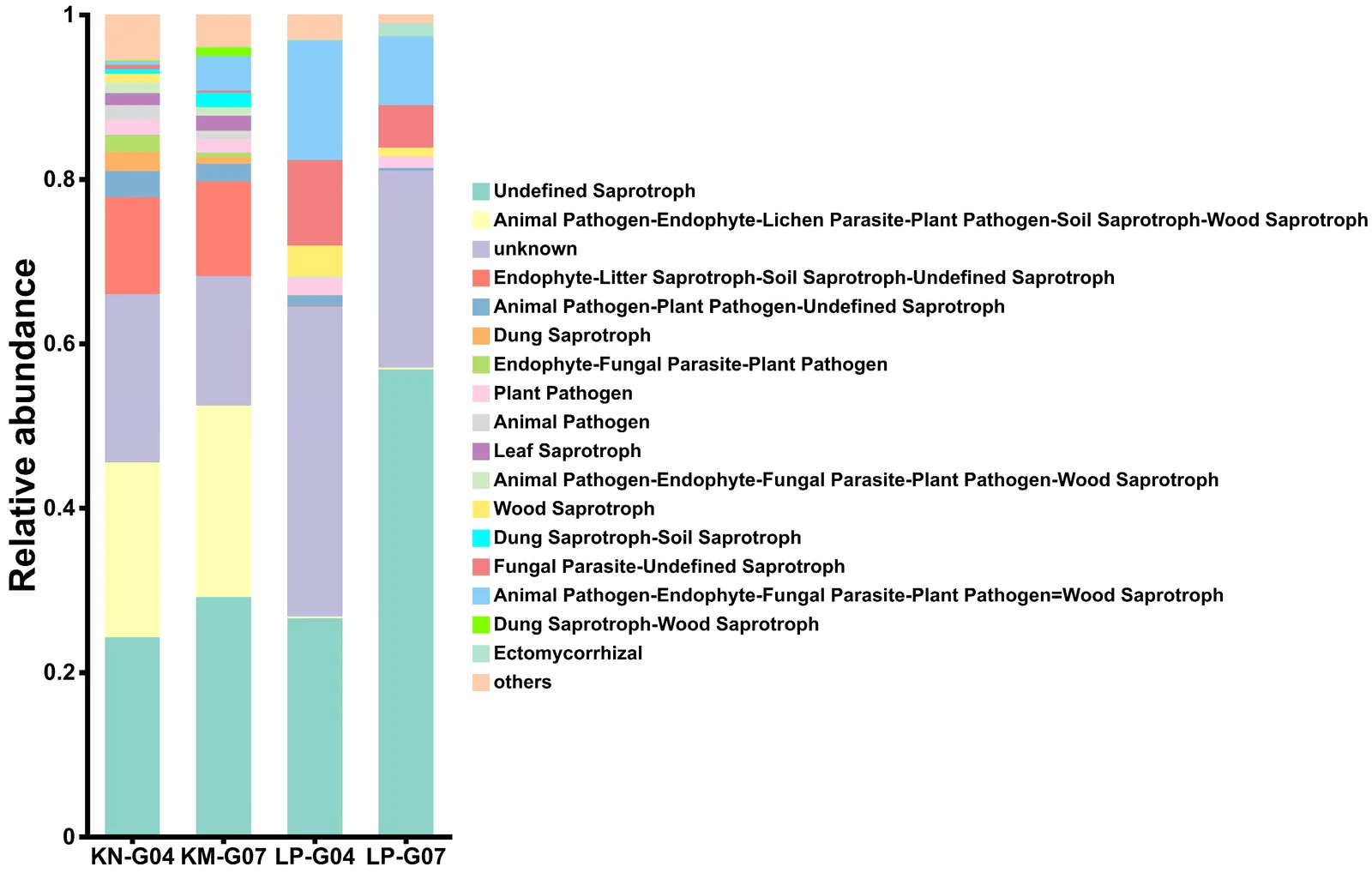
Predicted ecological functional guilds of rhizosphere fungal communities based on the FUNGuild database. KM-G04, KM-G07, LP-G04, and LP-G07 represent rhizosphere soil samples of ginger cultivars G04 and G07 grown at two planting locations, Kunming and Luoping, respectively.

Functional predictions revealed distinct variations in the microbial functional composition of the ginger rhizosphere across different growing locations and cultivars (Figure 6). Fungal functional profiling via FUNGuild (Figure 6) demonstrated marked compositional shifts in functional guilds among treatments. Undefined saprotroph represented a high proportion across all treatments, reaching its maximum in LP-G07. In KM-G04 and KM-G07, a complex multi-trophic guild—comprising animal pathogen–endophyte–lichen parasite–plant pathogen–soil saprotroph–wood saprotroph—showed higher relative abundances. Conversely, LP-G04 was dominated by unassigned functional guilds (unknown), followed by a notable proportion of the animal pathogen–endophyte–fungal parasite–plant pathogen–wood saprotroph guild. LP-G07 was mainly composed of undefined saprotroph and unknown. Overall, while fungal communities across all treatments encompassed saprotrophic, pathotrophic, and symbiotrophic mixed-trophic guilds, their relative abundance distributions varied substantially among treatment groups.

Functional predictions based on KEGG Orthology (KO) groups revealed distinct functional profiles between the Kunming and Luoping treatments, with several key categories showing pronounced location-specific enrichment patterns (Table S1 and Figure S1). Luoping exhibited significantly higher predicted abundances of glutathione S-transferase (K00799; involved in oxidative stress response), acetyl-CoA C-acetyltransferase (K00626; linked to fatty acid metabolism), and an LacI family transcriptional regulator (K02529; associated with carbon utilization regulation), along with branched-chain amino acid transport system components (K01996 and K01999, ATP-binding and substrate-binding proteins, respectively), as well as the iron complex outer membrane receptor protein (K02014; mediating siderophore-dependent iron uptake). In contrast, Kunming soils showed a significantly higher predicted abundance of UDP-glucose 4-epimerase (K01784), an enzyme involved in galactose metabolism and exopolysaccharide (EPS) biosynthesis.

### 2.7. Relationships Among Soil Properties, Enzyme Activities, Microbial Communities, and Ginger Yield

**Figure 7:**
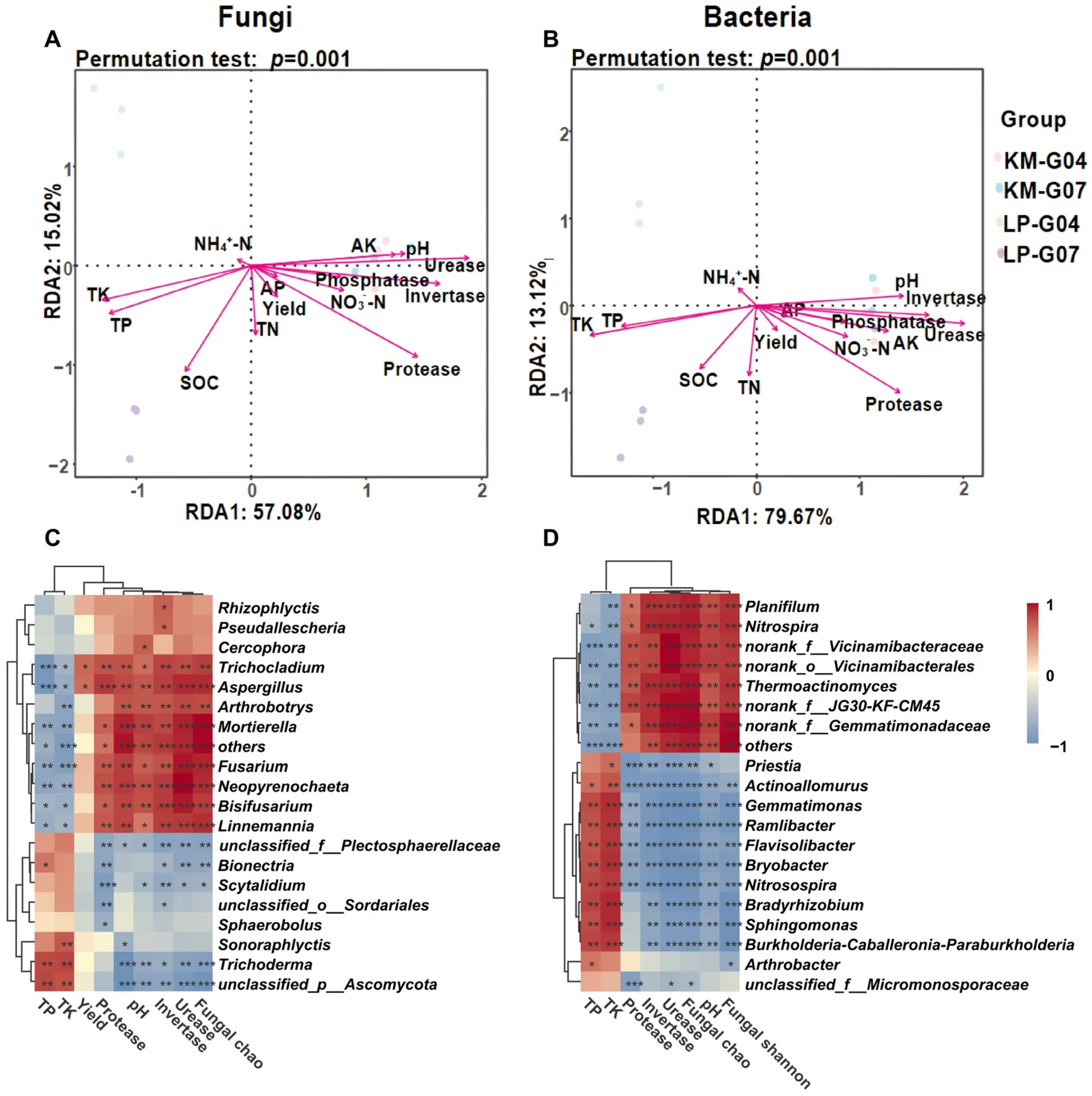
Associations among soil properties, enzyme activities, microbial communities, and ginger yield. (**A**,**B**) Redundancy analysis (RDA) of fungal (**A**) and bacterial (**B**) communities at the genus level. Permutation tests were used to assess model significance (*p* = 0.001). The dotted horizontal and vertical lines in panels (**A**) and (**B**) indicate the zero-reference lines for the RDA1 and RDA2 axes, respectively. (**C**,**D**) Spearman correlation heatmaps showing relationships among dominant fungal (**C**) and bacterial (**D**) genera, soil properties, enzyme activities, microbial diversity indices, and ginger yield. Purple and blue indicate positive and negative correlations, respectively. Asterisks indicate significance levels: * *p* < 0.05, ** *p* < 0.01, and *** *p* < 0.001.

Redundancy analysis (RDA) demonstrated that soil properties and enzyme activities significantly influenced rhizosphere microbial community compositions (Figure 7; *p* = 0.001). The first two RDA axes explained 72.10% and 92.79% of the total community variation for fungi and bacteria, respectively. Envfit analysis revealed that pH, TP, TK, AK, invertase, urease, and protease were all significantly correlated with both bacterial and fungal communities, while SOC was additionally significantly associated with the fungal community (Tables S2 and S3). Among these, urease, protease, and invertase exhibited strong explanatory power (*p* < 0.05).

**Figure 8:**
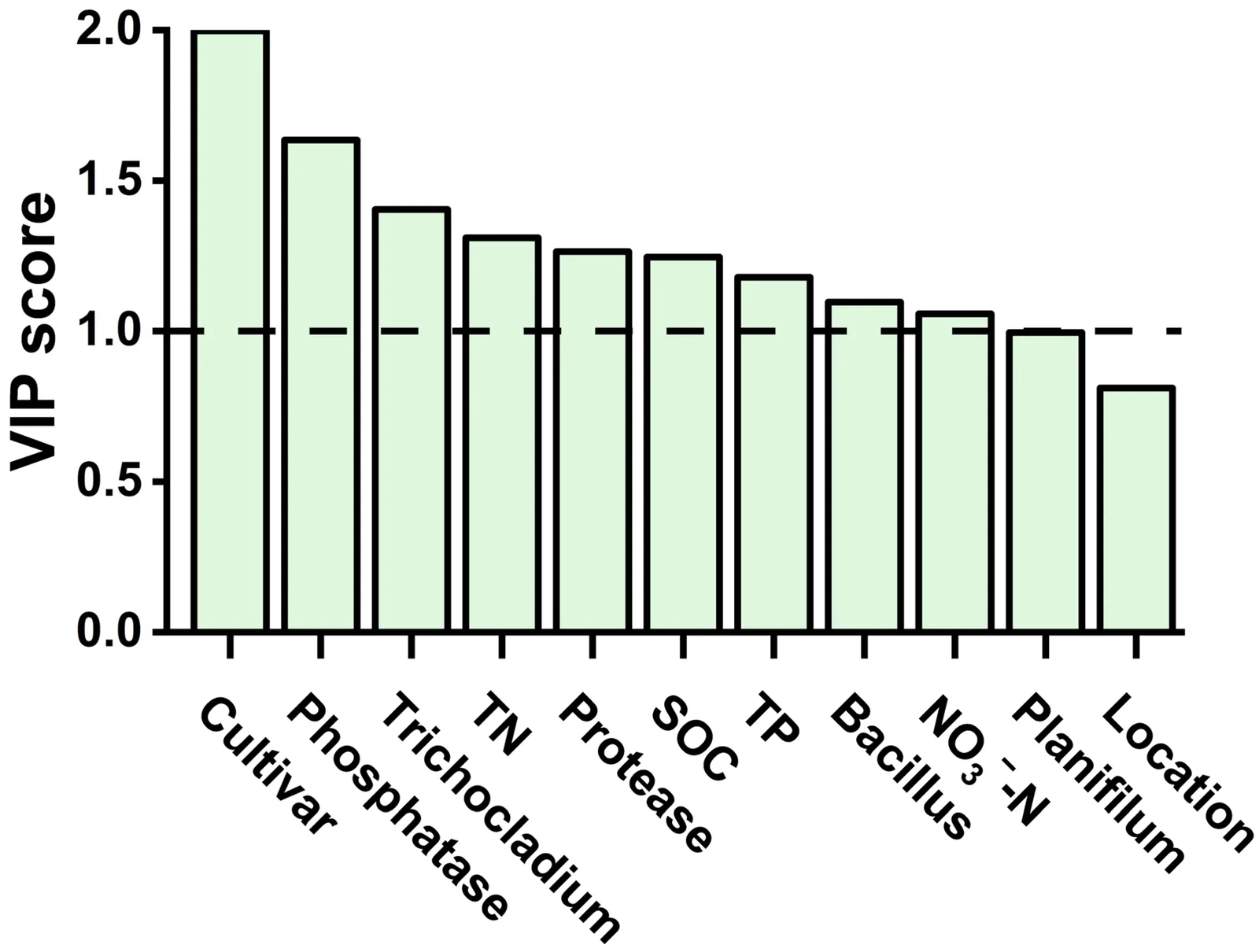
Variable importance in projection (VIP) scores derived from the partial least squares regression (PLSR) model for ginger yield. The dashed line indicates VIP = 1, and variables with VIP > 1 were considered important contributors to yield variation.

Spearman correlation analysis was performed to explore the relationships among soil properties, enzyme activities, alpha diversity, and dominant microbial genera (Figure 7C,D). For the fungal community (Figure 7C), the genera *Mortierella*, *Linnemannia*, *Trichocladium*, *Aspergillus*, *Fusarium*, *Bisifusarium*, and *Neopyrenochaeta* were generally positively correlated with enzyme activities, pH, and fungal alpha diversity but were negatively correlated with TP and TK. Conversely, *Trichoderma*, *unclassified__p__Ascomycota*, *unclassified__o__Sordariales*, and *Scytalidium* displayed opposite trends. Notably, ginger yield was significantly and positively correlated with *Trichocladium* and *Aspergillus*, whereas its correlations with most other fungal genera were relatively weak or nonsignificant (Figure 7C). For the bacterial community (Figure 7D), the genera *Planifilum*, *Nitrospira*, *Thermoactinomyces*, and several unclassified taxa were positively correlated with enzyme activities, pH, and microbial diversity. In contrast, *Gemmatimonas*, *Bryobacter*, *Bradyrhizobium*, *Sphingomonas*, and *Burkholderia–Caballeronia–Paraburkholderia* were positively correlated with TP and TK (Figure 7D). Partial least squares regression–variable importance in projection (PLSR-VIP) analysis was performed (Figure 8). Cultivar ranked highest in importance (VIP score > 1), followed by phosphatase, *Trichocladium*, TN, protease, SOC, TP, *Bacillus*, and NO_3_^−^-N.

## 3. Discussion

Although ginger yield was primarily affected by cultivar, rhizosphere microbial composition varied with both growing location and cultivar, suggesting that cultivar-related yield differences were accompanied by shifts in specific microbial taxa and predicted microbial functions. Seasonal variation and temperature can influence microbial community composition and functional potential, either directly by affecting microbial growth and activity or indirectly by altering soil moisture, nutrient availability, plant development, and root-derived carbon inputs [19,20,21]. However, all plants were grown during the same April–October growing season and sampled only at harvest, so the temporal seasonal dynamics of microbial communities could not be characterized. Although the two sites differ in elevation, their growing season temperature regimes are broadly comparable (Section 4.1). Therefore, temperature differences between locations are unlikely to serve as the primary driver for the observed divergence in rhizosphere microbial communities. Based on the present results, cultivar was the predominant factor affecting ginger yield, whereas growing location and its associated edaphic conditions were the major factors shaping rhizosphere microbial communities. In particular, RDA identified soil pH, TP, TK, invertase, urease, and protease as the main measured environmental variables associated with bacterial and fungal community variation. These findings indicate that ginger productivity is closely associated with the combined effects of cultivar identity, location-dependent soil biochemical conditions, and rhizosphere microbial community structure, thereby supporting our initial hypothesis regarding the interplay between host genotype and environmental factors. In the following sections, we first discuss how these cultivar–location combinations modulate soil biochemical status, subsequently analyze the assembly and predicted functions of rhizosphere microbial communities, and finally examine the links between specific microbial taxa and ginger yield.

### 3.1. Cultivar and Location Jointly Influenced Ginger Yield and Soil Properties

In the present study, ginger yield was dictated by both cultivar identity and planting location, with the significant cultivar × location interaction underscoring that genotypic plasticity is intrinsically linked to the environmental context. The superior agronomic phenotype of G07, particularly at the Kunming site, confirms that, while cultivar identity represents the primary determinant of yield potential, this potential is fundamentally modulated by the planting environment. The edaphic interface serves as the foundational substrate for this interaction, as physicochemical properties directly govern nutrient stoichiometry, root architecture, and microbial metabolic fluxes [4,5].

The distinct soil properties observed at the two locations—with higher pH and available potassium (AK) in Kunming, and higher total phosphorus (TP) and potassium (TK) in Luoping—established distinct edaphic templates. These site-specific soil conditions were pivotal in modulating the rhizosphere microbiome, a pattern consistent with the “soil reservoir, plant filter” paradigm, where soil acts as the primary microbial seed bank, and host genotype exerts a selective filter on community assembly [14,15,16]. Consequently, the observed yield patterns reflect a synergetic mechanism: the cultivar acts as a selective gatekeeper for microbial recruitment, while location-specific soil properties provide the environmental framework within which this niche colonization proceeds.

Furthermore, the higher soil organic carbon (SOC) and total nitrogen (TN) contents associated with G07 suggest that cultivar-specific root–soil interactions may actively reconfigure rhizosphere carbon and nitrogen cycling. While this likely arises from cultivar-specific heterogeneity in root biomass, exudate metabolic profiles, or nutrient uptake strategies, these mechanisms remain speculative given the lack of direct root physiological measurements. Future research integrating root trait analysis, metabolomic profiling of root exudates, and microbial functional genomics will be essential to fully elucidate the specific feedback loops through which ginger cultivars orchestrate rhizosphere biogeochemical processes to enhance productivity.

### 3.2. Soil Enzyme Activities Indicate Differences in Nutrient Cycling Potential

Soil enzyme activities exhibited pronounced heterogeneity across the four cultivar–location combinations, with KM treatments consistently maintaining higher activities of invertase, urease, protease, and phosphatase than LP treatments. Among all experimental groups, KM-G07 exhibited the highest enzymatic capacity, suggesting stronger soil biochemical activity. As essential indicators of microbial metabolism and biogeochemical processes, these enzymes facilitate critical transformation pathways: invertase participates in carbon mineralization, urease and protease are involved in nitrogen turnover, and phosphatase catalyzes phosphorus mobilization [7,17,18]. Consequently, the enhanced enzymatic profile of KM-G07 may reflect greater nutrient-cycling potential and a more favorable biochemical environment for ginger growth.

These results align with established findings in ginger cultivation research. Previous studies have demonstrated that the application of plant growth-promoting rhizobacteria (PGPR) and balanced fertilizers can enhance soil biochemical activity [22]. Similarly, interventions such as biochar application or mineral fertilization have been shown to modulate the rhizosphere enzymatic landscape and ginger growth [23,24]. In the present study, the concurrence of high enzymatic activity and maximum crop yield in KM-G07 suggests that enzyme-mediated nutrient cycling may be an important contributor to ginger productivity.

Notably, our findings reveal a partial dissociation between total soil nutrient stocks and biological activity. Although LP soils contained higher total phosphorus (TP) and total potassium (TK), these nutrient pools did not correspond to higher enzymatic activity or yield than in KM. This pattern indicates that total nutrient contents alone may not adequately represent nutrient availability to plants. Instead, nutrient bioavailability, which is closely linked to enzyme-mediated transformation and microbial activity, may be more relevant to nutrient acquisition. This underscores the importance of integrating both chemical and biological indicators when evaluating soil fertility and ginger production potential.

### 3.3. Rhizosphere Microbial Communities Were Mainly Shaped by Growing Location and Soil Conditions

Alpha and beta diversity metrics revealed that rhizosphere bacterial and fungal community assembly was primarily differentiated by planting location rather than cultivar, underscoring the overarching influence of edaphic heterogeneity. Both fungal and bacterial Chao1 and Shannon indices were higher in KM than in LP, while PCoA based on Bray–Curtis distances exhibited a clear clustering pattern by site. This indicates that site-specific soil conditions acted as an important environmental filter on the rhizosphere microbiome. This is consistent with established ecological paradigms proposing that soil pH, nutrient stoichiometry, and soil physical structure serve as primary determinants of microbial community composition [25,26]. Specifically, soil pH has been widely recognized as a pivotal factor shaping bacterial community structure across diverse terrestrial ecosystems.

Taxonomic profiling further corroborated this location-dependent differentiation. At the phylum level, *Ascomycota* remained the predominant fungal group, while *Mortierellomycota* showed a distinct enrichment in KM. The bacterial community exhibited significant shifts in the relative abundance of *Pseudomonadota*, *Actinomycetota*, *Bacillota*, *Acidobacteriota*, and *Chloroflexota* between KM and LP. These taxonomic shifts reflect niche differentiation driven by site-specific differences in pH, nutrient availability, and enzymatic activity. The rhizosphere represents a high-activity interface where root–soil–microbe interactions govern nutrient cycling and plant resilience [27,28]. Consequently, our findings imply that ginger rhizosphere assembly was not solely a product of host genotype selection but was instead mediated by the synergistic interplay between soil environmental filtering and plant-mediated recruitment. At the genus level, several taxa exhibited contrasting distribution patterns. The enrichment of *Trichoderma* in LP is noteworthy, given its well-established role in pathogen suppression, nutrient mobilization, and root-associated beneficial interactions [29]. However, the fact that LP did not exhibit the highest ginger yield suggests an uncoupling between the abundance of putatively beneficial genera and phenotypic output. Similarly, while *Fusarium* was more abundant in KM (the higher-yielding site), genus-level sequencing lacks the taxonomic resolution to distinguish between pathogenic and non-pathogenic or saprotrophic strains. Therefore, these taxa should be viewed as indicator species for rhizosphere community shifts rather than direct causal agents of yield variation. Future work utilizing species-level phylogenetics, pathogen isolation, and functional meta-transcriptomics will be required to elucidate the precise ecological roles that these taxa play in ginger production.

Functional predictions suggested that location- and cultivar-associated shifts in microbial community composition were accompanied by distinct changes in microbial functional potential. The higher predicted abundances of glutathione S-transferase (K00799), acetyl-CoA C-acetyltransferase (K00626), and the LacI family transcriptional regulator (K02529) in the Luoping treatments may reflect enhanced potential for oxidative stress responses, carbon utilization, and metabolic regulation. The enrichment of branched-chain amino acid transport system components (K01996 and K01999) further indicates a greater predicted capacity for ATP-dependent nutrient acquisition, whereas the higher abundance of the iron complex outermembrane receptor protein (K02014), particularly in LP-G07, suggests location- and cultivar-specific adaptation of bacterial iron acquisition strategies. Bacterial stress response systems and ABC transporters play important roles in coordinating environmental adaptation and the uptake or efflux of nutrients and metabolites [30,31]. In contrast, the higher predicted abundance of UDP-glucose 4-epimerase (K01784) in the Kunming treatments indicates enhanced potential for galactose metabolism and exopolysaccharide biosynthesis, which may contribute to microbial adaptation to the local rhizosphere environment. FUNGuild analysis further revealed that saprotrophic, pathotrophic, symbiotrophic, and complex multi-trophic guilds occurred across all treatments, but their relative distributions differed markedly. Undefined saprotrophs and unassigned guilds were particularly abundant in LP-G07, whereas complex multi-trophic guilds showed relatively high abundances in the Kunming treatments. Similar shifts in saprotrophic and mixed-trophic fungal guilds have been reported in continuously cropped rhizosphere soils and are commonly associated with variations in soil properties [32]. Nevertheless, because PICRUSt2 and FUNGuild infer functional profiles from marker gene sequences and taxonomic assignments, respectively, these results represent predicted functional potential rather than directly measured metabolic activity [33,34].

### 3.4. Key Soil Biochemical and Microbial Indicators Associated with Ginger Yield

RDA further elucidated that pH, TP, TK, and key enzymatic activities (invertase, urease, and protease) act as the principal environmental drivers of bacterial and fungal community variation. These variables define two fundamental ecological gradients within the ginger rhizosphere: soil physicochemical status and enzyme-mediated nutrient turnover. The higher explanatory power observed for bacterial communities implies that bacterial taxa exhibit greater niche sensitivity to soil nutrient fluctuations than their fungal counterparts, which are often more strongly regulated by complex organic substrates and long-term soil developmental processes [25].

Our integrative analysis, employing both Spearman correlation heatmaps and PLSR-VIP, successfully synthesized the complex relationships between soil properties, enzyme activities, microbial assemblages, and crop yield. The high VIP score of “cultivar” confirms that genetic background acts as the primary determinant of yield potential. Among biochemical indicators, phosphatase, TN, protease, SOC, TP, and NO_3_^−^-N displayed VIP scores > 1, corroborating that nitrogen and phosphorus cycling are critical determinants of ginger productivity. These findings align with the prevailing view that soil enzyme-mediated nutrient transformation serves as a key indicator of soil biological functioning and crop performance [7,17,22,29]. Previous studies in ginger systems have similarly reported that fertilizer or biochar-induced optimization of the rhizosphere biochemical landscape is essential for enhancing rhizome biomass [22,23,24].

Regarding microbial predictors, *Bacillus* and *Trichocladium* emerged as key variables with VIP scores > 1. *Bacillus* species are widely recognized for their rhizosphere competence and multifunctional roles in nutrient solubilization, phytohormone production, and biocontrol [35]. Their significant contribution in our VIP model likely reflects their active involvement in nutrient-related rhizosphere processes. Similarly, *Trichocladium* exhibited a positive correlation with yield; however, given the current lack of functional data regarding *Trichocladium* in ginger-specific rhizospheres, its role should be treated as an ecological indicator rather than a functionally validated agent of yield improvement. This highlights the inherent taxonomic–functional ambiguity of amplicon sequencing, where a high relative abundance does not necessarily equate to proportional functional contribution.

Synthetically, these results suggest that ginger yield variation is not a product of single-factor modulation but rather an emergent property of the tripartite plant–soil–microbiome interaction [28]. From an agronomic perspective, the superior yield and enhanced enzymatic activity observed in KM-G07 imply that coupling high-performing cultivars with specific soil edaphic environments—thereby optimizing nutrient cycling—is a promising strategy for sustainable ginger production. Nevertheless, the present study included only two ginger cultivars and two growing locations, which may limit the generalizability of the findings to other cultivars and production environments. Moreover, because growing location represents the combined effects of soil properties, climatic conditions, and other environmental factors, the independent contributions of these factors could not be fully disentangled. Future studies should therefore incorporate additional cultivars, locations, and growing seasons, together with controlled multi-factor experiments, microbial isolation, metagenomic functional profiling, and gnotobiotic field validation, to establish direct causal links between specific microbial taxa and ginger yield enhancement.

## 4. Materials and Methods

### 4.1. Experimental Design and Growth Conditions

**Figure 9:**
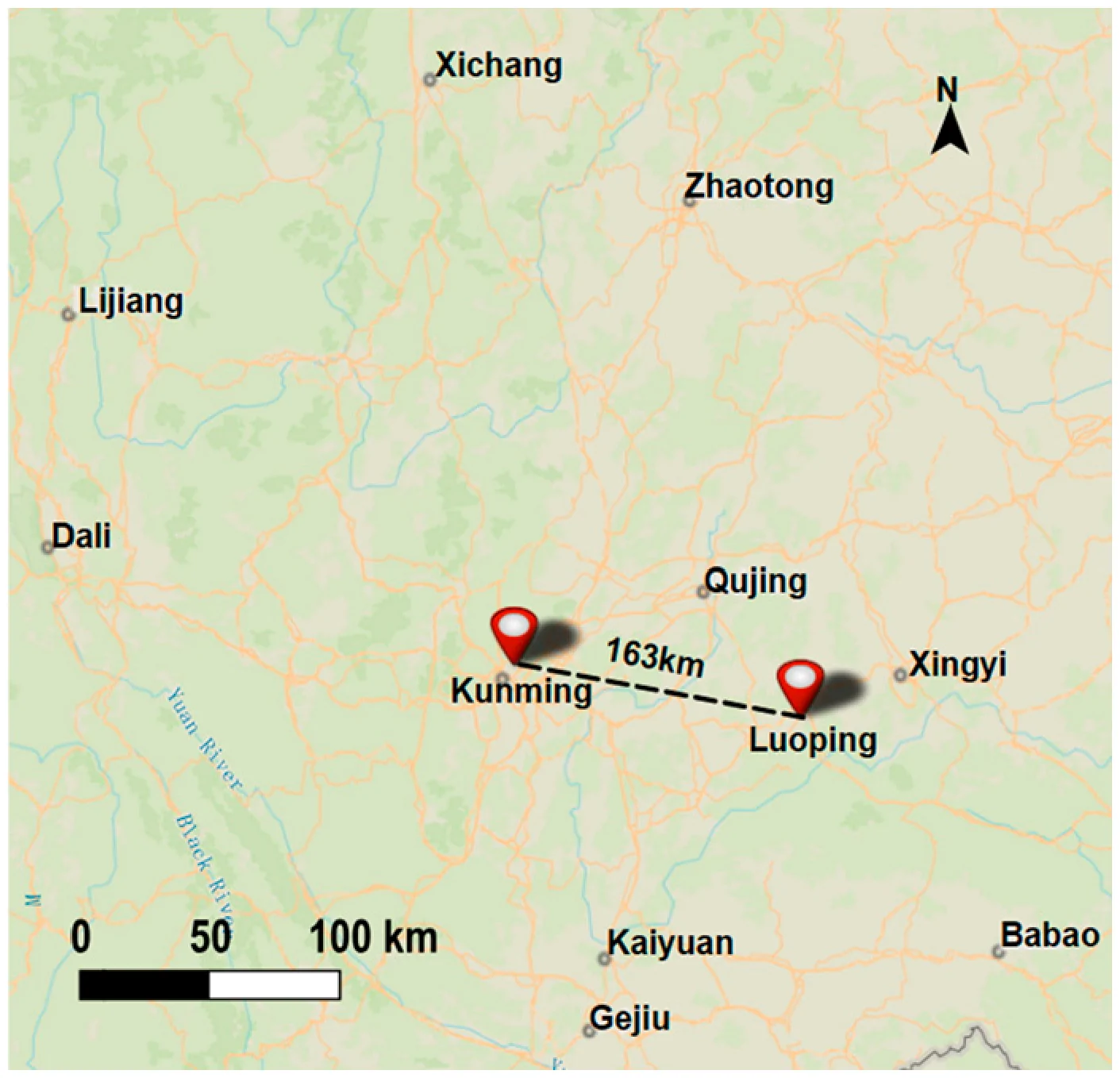
Geographic locations of the two experimental sites in Yunnan Province, China. The two sites are approximately 163 km apart. The map was generated using QGIS (version 3.44.14), and the base map shows the provincial boundary of Yunnan Province.

The experiment was conducted at the experimental field base of Yunnan Agricultural University in Kunming (KM; 102°44′56.12″ E, 25°07′41.75″ N; 1904 m a.s.l.) and a planting demonstration site in Luoping, Qujing (LP; 104°29′47.64″ E, 24°57′45.45″ N; 1457 m a.s.l.), Yunnan Province, China. The two sites are approximately 163 km apart and lie within the subtropical plateau monsoon climate zone (Figure 9). Although their elevations differ, the two locations experience broadly comparable air temperature regimes across the April–October ginger-growing season. The sites were selected during the experimental planning stage because Luoping is a major production and distribution area for the geographical indication product “Luoping Xiaohuangjiang”, whereas Kunming provided a second representative growing environment for multi-location cultivar evaluation.

Two ginger cultivars, ‘Dianjiang G04’ (G04) and ‘Dianjiang G07’ (G07), were used in this study. These two cultivars were selected from seven ginger germplasm accessions that were evaluated under comparable cultivation conditions in a preliminary screening trial conducted in 2024 in both Kunming and Luoping. G04 exhibited the lowest rhizome yield, while G07 exhibited the highest, making them ideal materials for investigating whether cultivar-associated differences in productivity are accompanied by shifts in soil biochemical properties and rhizosphere microbial communities. The soil at both sites was classified as red soil. Before planting, bulk soil samples were collected from the 0–30 cm plow layer, air-dried, and passed through a 1 mm sieve for the determination of initial soil properties. The initial soil properties at KM were as follows: pH 6.95, SOC 11.20 g kg^−1^. available nitrogen (AN) 72.89 mg kg^−1^, total phosphorus (TP) 2.87 g kg^−1^, total potassium (TK) 9.84 g kg^−1^, available potassium (AK) 210.19 mg kg^−1^, available phosphorus (AP) 150 mg kg^−1^, and alkali-hydrolyzable nitrogen (AN) 97.77 mg kg^−1^. The corresponding values at LP were: pH 6.15, SOC 12.20 g kg^−1^, AN 63.17 mg kg^−1^, TP 2.71 g kg^−1^, and TK 20.71 g kg^−1^. AK 190.8 mg kg^−1^, AP 140 mg kg^−1^, and AN 91.17 mg kg^−1^.

### 4.2. Plant Harvest and Sampling Processing

Ginger was planted in April and harvested at the end of the growing season in October, when destructive sampling was conducted. During sampling, three ginger plants with uniform growth were randomly selected from each treatment. The roots were gently shaken to remove loosely attached soil, and the soil tightly adhered to the root surface was collected as rhizosphere soil. Rhizosphere soil samples from the three plants within the same treatment were thoroughly mixed to form one composite sample. The collected soil samples were divided into three subsamples: approximately 5 g was immediately frozen in liquid nitrogen and stored at −80 °C for subsequent microbial DNA extraction (see Section 4.5 for DNA extraction details); approximately 50 g was stored at 4 °C for the determination of soil enzyme activities and inorganic nitrogen (NH_4_^+^-N and NO_3_^−^-N); and the remaining soil was air-dried, ground, and passed through a 1 mm sieve for the analysis of soil physicochemical properties. Ginger yield was calculated based on the fresh weight of harvested edible rhizomes and expressed as kg mu^−1^ (1 mu = 1/15 ha).

### 4.3. Soil Physicochemical Analysis

The fresh soil subsample stored at 4 °C was used for the determination of NH_4_^+^-N, NO_3_^−^-N, and enzyme activities, whereas the air-dried soil fraction was used for the determination of pH, soil organic carbon (SOC), total nitrogen (TN), total phosphorus (TP), total potassium (TK), available phosphorus (AP), and available potassium (AK). Soil pH was measured potentiometrically using a pH meter in a 1:2.5 (*w*/*v*) soil:water suspension [36]. For the determination of TN, TP, and TK, the soil samples were digested with an H_2_SO_4_–HClO_4_ mixture prior to analysis [37]. After digestion, the extracts were cooled, diluted to a fixed volume, and analyzed using an HM–GT4 soil nutrient analyzer (Shandong Hengmei Electronic Technology Co., Ltd., Weifang, China) according to the manufacturer’s protocol. Soil AP, AK, NO_3_^−^-N, NH_4_^+^-N, and SOC were determined using the corresponding extraction, oxidation, color development, and colorimetric procedures according to the manufacturer’s instructions. The initial soil samples were analyzed using the same method.

### 4.4. Determination of Soil Enzyme Activities

Soil enzyme activities were determined according to the methods described by Guan [38], with minor modifications. All assays were conducted in triplicate using fresh soil samples, and enzyme activities were expressed per gram of oven-dry soil. Invertase activity was determined by measuring the reducing sugars released after incubation with sucrose using the 3,5-dinitrosalicylic acid (DNS) colorimetric method. Urease activity was estimated by quantifying the ammonium nitrogen produced from urea hydrolysis using sodium phenol and sodium hypochlorite. Protease activity was determined based on the amount of tyrosine released from casein during incubation. Phosphatase activity was measured by quantifying the phenol released from disodium phenyl phosphate using 4-aminoantipyrine.

### 4.5. DNA Extraction, PCR Amplification, and Sequencing

Total microbial genomic DNA was extracted from the soil samples using an E.Z.N.A.^®^ Soil DNA Kit (Omega Bio-tek, Norcross, GA, USA) according to the manufacturer’s instructions. The detailed extraction protocol is available at: https://omegabiotek.com/product/soil-dna-extraction-kit-e-z-n-a-soil-dna-kit/ (15 September 2026). The quality and concentration of the DNA were determined by 1.0% agarose gel electrophoresis and a NanoDrop2000 spectrophotometer (Thermo Fisher Scientific, Waltham, MA, USA), and it was kept at −80 °C prior to further use. The V3–V4 hypervariable region of the bacterial 16S rRNA gene was amplified with primer pairs 338F (5′-ACTCCTACGGGAGGCAGCAG-3′) and 806R (5′-GGACTACHVGGGTWTCTAAT-3′). The fungal internal transcribed spacer 1 (ITS1) region was amplified with the primers ITS1F (5′-CTTGGTCATTTAGAGGAAGTAA-3′) and ITS2R (5′-GCTGCGTTCTTCATCGATGC-3′) by a T100 Thermal Cycler PCR thermocycler (Bio-Rad Laboratories, Hercules, CA, USA). The PCR reaction mixture included 4 μL 5 × Fast Pfu buffer, 2 μL 2.5 mM dNTPs, 0.8 μL of each primer (5 μM), 0.4 μL Fast Pfu polymerase, 10 ng of template DNA, and ddH_2_O to a final volume of 20 µL. The PCR amplification cycling conditions were as follows: initial denaturation at 95 °C for 3 min, followed by 27 cycles of denaturing at 95 °C for 30 s, annealing at 55 °C for 30 s and extension at 72 °C for 45 s, a single extension at 72 °C for 10 min, and ending at 4 °C. The PCR product was extracted from 2% agarose gel, purified using a PCR Clean-Up Kit (YuHua, Shanghai, China) according to the manufacturer’s instructions, and quantified using Qubit 4.0 (Thermo Fisher Scientific, Waltham, MA, USA). The purified products were pooled in equimolar amounts, and a DNA library was constructed using an SMRTbell prep kit 3.0 (Pacific Biosciences, Menlo Park, CA, USA) according to PacBio’s instructions. Purified SMRTbell libraries were sequenced on the Pacbio Sequel IIe System (Pacific Biosciences, Menlo Park, CA, USA) by Majorbio Bio-Pharm Technology Co., Ltd. (Shanghai, China). High-fidelity (HiFi) reads were obtained from the subreads, generated using circular consensus sequencing via SMRT Link v11.0.

### 4.6. Bioinformatic Processing

Raw sequencing reads were demultiplexed according to their barcodes and quality-filtered. For bacterial 16S rRNA gene sequencing, the V3–V4 hypervariable region was targeted using the 338F/806R primer pair, yielding 1,273,664 optimized sequences across 24 samples (average length: 417 bp). For fungal ITS sequencing, the ITS1 region was targeted using the ITS1F/ITS2R primer pair, yielding 1,858,490 optimized sequences across 24 samples (average length: 246 bp). High-quality sequences were subsequently clustered into operational taxonomic units (OTUs) at 97% sequence similarity using UPARSE 7.1 [39,40]. To minimize sequencing depth bias, all samples were rarefied to the minimum sequencing depth prior to diversity analyses.

Taxonomic annotation was performed using the RDP Classifier v2.2 [41], with a confidence threshold of 0.7. Bacterial 16S rRNA gene OTUs were classified against the SILVA database (v138), whereas fungal ITS OTUs were annotated against the UNITE database (v8.0). After filtering for OTUs with at least 5 sequence counts in at least three samples, 2605 bacterial and 897 fungal OTUs were retained. Taxonomic assignments ranged from kingdom to species level. Based on the filtered OTU table, alpha diversity, beta diversity, community composition, and environmental association analyses were performed. Differentially abundant bacterial and fungal taxa among treatments were identified using linear discriminant analysis effect size (LEfSe), with an LDA score threshold of 4.0. Bacterial functional profiles were predicted using PICRUSt2, whereas fungal ecological guilds were assigned using FUNGuild.

### 4.7. Statistical Analysis

Soil microbial community composition and diversity were analyzed using specialized bioinformatics platforms (Majorbio Cloud; Wekemo Bioincloud). Alpha diversity (Chao1 and Shannon indices) and beta diversity (Bray–Curtis PCoA, PERMANOVA) were assessed to characterize community structure. To elucidate the relationships between environmental drivers and microbial communities, redundancy analysis (RDA) was performed using environmental variables pre-screened by envfit (pH, TP, TK, and key enzyme activities). Ginger yield was excluded from the RDA as a response variable.

Spearman correlation analysis and PLSR-VIP (SPSSPRO, VIP > 1) were subsequently employed to identify key microbial genera and soil biochemical indicators associated with ginger yield, with variables standardized via z-score transformation. Differences in ginger yield, soil physicochemical properties, and enzyme activities were evaluated using two-way ANOVA followed by Tukey’s post hoc test (*p* < 0.05) in SPSS(version 29.0.2.0; IBM Corp., Armonk, NY, USA). All figures and tables were prepared using Origin 2024b and Microsoft Excel 2021.

## 5. Conclusions

This study demonstrated that ginger yield and rhizosphere characteristics were jointly influenced by cultivar and growing location, with cultivar exerting the strongest effect on yield. G07 consistently produced higher yields than G04, and KM-G07 achieved the highest yield. Compared with Luoping, Kunming generally exhibited higher soil pH, available potassium, enzyme activities, and microbial alpha diversity. The bacterial and fungal communities were clearly differentiated between the two locations, with cultivar–level segregation also detectable within each location—particularly in Luoping, where edaphic conditions (lower pH and higher NO_3_^−^-N, AP, and AK) appeared to amplify cultivar-specific microbial recruitment. RDA revealed that pH, TP, TK, invertase, urease, and protease were closely associated with microbial community variation. Functional prediction further revealed location- and cultivar-dependent differences in bacterial metabolic potential and fungal ecological guild composition. Integrated correlation and PLSR-VIP analyses identified cultivar, soil nutrients, enzyme activities, *Trichocladium*, and *Bacillus* as important variables associated with ginger yield. Collectively, these findings demonstrate that high-yielding cultivars do not rely on a universally beneficial microbial community across different soil environments; instead, they deploy location-specific microbial recruitment strategies that optimize performance under local edaphic conditions. From an agronomic perspective, matching high-yielding cultivars with suitable soil environments—and actively managing rhizosphere nutrient cycling through enzyme activity modulation—may represent an effective strategy for sustainable ginger production. Future studies should explore the mechanistic basis of cultivar-specific microbial recruitment, particularly the role of root exudates in shaping rhizosphere communities under contrasting soil conditions, and validate these findings through long-term field trials across broader geographic scales.

## Figures and Tables

**Table 1:** Soil physicochemical properties in rhizosphere of two ginger cultivars (G04, G07) grown in Kunming (KM) and Luoping (LP), respectively.

Treatment	pH	SOC (g kg^−1^)	TN (g kg^−1^)	TP (g kg^−1^)	TK (g kg^−1^)	NH_4_^+^-N (mg kg^−1^)	NO_3_^−^-N (mg kg^−1^)	AP (mg kg^−1^)	AK (mg kg^−1^)
KM-G04	8.00 ± 0.56 a	10.79 ± 0.33 b	2.40 ± 0.21 b	2.17 ± 0.09 b	8.66 ± 0.19 b	33.03 ± 7.05 a	55.52 ± 7.23 a	165.87 ± 19.91 a	364.49 ± 23.88 a
LP-G04	6.32 ± 0.30 b	11.16 ± 0.52 b	2.30 ± 0.08 b	2.48 ± 0.13 a	19.82 ± 3.67 a	38.54 ± 3.86 a	39.25 ± 4.84 b	142.33 ± 17.27 a	271.97 ± 41.66 b
KM-G07	7.71 ± 0.32 a	11.87 ± 0.60 b	2.80 ± 0.21 a	1.28 ± 0.09 c	10.09 ± 1.84 b	30.62 ± 6.04 a	69.25 ± 10.40 a	188.54 ± 34.95 a	378.16 ± 21.85 a
LP-G07	6.20 ± 0.40 b	13.19 ± 0.17 a	2.97 ± 0.09 a	2.90 ± 0.13 a	23.06 ± 3.39 a	33.13 ± 5.13 a	46.94 ± 5.99 a	159.68 ± 11.83 a	274.49 ± 16.08 b
Location (L)	**	ns	ns	***	**	ns	*	ns	**
Cultivar (C)	ns	**	**	ns	ns	ns	ns	ns	ns
L × C	ns	ns	ns	***	ns	ns	ns	ns	ns

Data are presented as the mean ± SE (*n* = 3). Different lowercase letters (a, b, and c) within the same column indicate significant differences among treatments. SOC, soil organic carbon; TN, total nitrogen; TP, total phosphorus; TK, total potassium; NH_4_^+^-N, ammonium nitrogen; NO_3_^−^-N, nitrate nitrogen; AP, available phosphorus; AK, available potassium; L, location; C, cultivar; L × C, interaction between location and cultivar; ns, not significant. Asterisks indicate significance levels: * *p* < 0.05, ** *p* < 0.01, and *** *p* < 0.001.

## Data Availability

The raw amplicon sequencing data for bacterial 16S rRNA genes and fungal ITS regions generated in this study have been deposited in the NCBI Sequence Read Archive (SRA) under BioProject accession numbers PRJNA1515063 and PRJNA1515173, respectively. Submission and processing of the corresponding fungal ITS sequencing data are currently in progress, and these data will be linked to the same BioProject before publication. All other data supporting the findings of this study are available within the article and its Supplementary Information.

## Supplementary Materials

The following supporting information can be downloaded at: https://www.mdpi.com/article/10.3390/ijms27188428/s1.
