# Supplementary material for: Cultivar and Soil Properties Jointly Shape the Rhizosphere Microbial Communities and Biochemical Properties Associated with Ginger (Zingiber officinale) Productivity
Source: Int J Mol Sci. 2026 Sep 21;27(18):8428. doi: 10.3390/ijms27188428 (PMC13607680; doi:10.3390/ijms27188428)
Supplement: Supplementary file 1 [file ijms-27-08428-s001.zip › ijms-4545399-supplementary.pdf]

Table S1. Functional classification of the top 30 predicted bacterial KEGG orthologs.

KO_ID	KO_Description	KO_ID
K00059	3-oxoacyl-[acyl-carrier protein] reductase [EC:1.1.1.100]	K00059
K00128	aldehyde dehydrogenase (NAD+) [EC:1.2.1.3]	K00128
K00384	thioredoxin reductase (NADPH) [EC:1.8.1.9]	K00384
K00626	acetyl-CoA C-acetyltransferase [EC:2.3.1.9]	K00626
K00799	glutathione S-transferase [EC:2.5.1.18]	K00799
K01652	acetolactate synthase I/II/III large subunit [EC:2.2.1.6]	K01652
K01784	UDP-glucose 4-epimerase [EC:5.1.3.2]	K01784
K01897	long-chain acyl-CoA synthetase [EC:6.2.1.3]	K01897
K01952	phosphoribosylformylglycinamidine synthase [EC:6.3.5.3]	K01952
K01990	ABC-2 type transport system ATP-binding protein	K01990
K01992	ABC-2 type transport system permease protein	K01992
K01996	branched-chain amino acid transport system ATP-binding protein	K01996
K01999	branched-chain amino acid transport system substrate-binding protein	K01999
K02003	putative ABC transport system ATP-binding protein	K02003
K02004	putative ABC transport system permease protein	K02004
K02014	iron complex outermembrane receptor protein	K02014
K02015	iron complex transport system permease protein	K02015
K02016	iron complex transport system substrate-binding protein	K02016
K02032	peptide/nickel transport system ATP-binding protein	K02032
K02033	peptide/nickel transport system permease protein	K02033
K02034	peptide/nickel transport system permease protein	K02034
K02035	peptide/nickel transport system substrate-binding protein	K02035
K02529	LacI family transcriptional regulator	K02529
K03088	RNA polymerase sigma-70 factor, ECF subfamily	K03088
K03406	methyl-accepting chemotaxis protein	K03406
K03496	chromosome partitioning protein	K03496
K03704	cold shock protein	K03704
K06147	ATP-binding cassette, subfamily B, bacterial	K06147
K07090	uncharacterized protein	K07090
K08884	serine/threonine protein kinase, bacterial [EC:2.7.11.1]	K08884

Table S2. Envfit results for fungal genus-level RDA.

	RDA1	RDA2	r^2	p value
Yield	0.582568457	-0.812781639	0.19776654	0.378810595
pH	0.995845227	0.091061978	0.684273032	0.005497251
SOC	-0.473644692	-0.880716019	0.618605941	0.012493753
TN	0.054699033	-0.998502887	0.356447546	0.153923038
TP	-0.931550676	-0.36361152	0.67775613	0.014492754
TK	-0.966005954	-0.25851982	0.685289264	0.008995502
NH_4^+-N	-0.890641746	0.454705708	0.06829629	0.712143928
NO_3^--N	0.95512929	-0.296189195	0.429632669	0.084957521
AP	0.897637751	-0.44073401	0.128214001	0.549725137
AK	0.995909112	0.090360609	0.642802566	0.010994503
Invertase	0.993828936	-0.110923601	0.841393054	0.0009995
Urease	0.999137978	0.04151267	0.966101748	0.00049975
Protease	0.842989358	-0.537930239	0.874547694	0.0009995
Phosphatase	0.989817914	-0.142339375	0.39988917	0.089455272

Table S3. Envfit results for bacterial genus-level RDA.

	RDA1	RDA2	r^2	p value
Yield	0.568911037	-0.822399071	0.164772729	0.430784608
pH	0.996995298	0.077462089	0.674710064	0.006996502
SOC	-0.599593098	-0.800305015	0.430530233	0.082458771
TN	-0.089378668	-0.995997718	0.383688035	0.111444278
TP	-0.983670616	-0.179978109	0.627640294	0.010994503
TK	-0.978212925	-0.207604126	0.778265842	0.0009995
NH_4^+-N	-0.662095481	0.749419491	0.125500665	0.549225387
NO_3^--N	0.926699884	-0.375802241	0.45046568	0.073963018
AP	0.924636108	-0.380851767	0.152616921	0.47876062
AK	0.974157471	-0.225869923	0.619857634	0.018490755
Invertase	0.997818761	-0.066013036	0.791142888	0.002498751
Urease	0.994656945	-0.10323547	0.958217576	0.0009995
Protease	0.810076027	-0.58632485	0.810466664	0.0009995
Phosphatase	0.976832073	-0.214007247	0.416464575	0.084457771

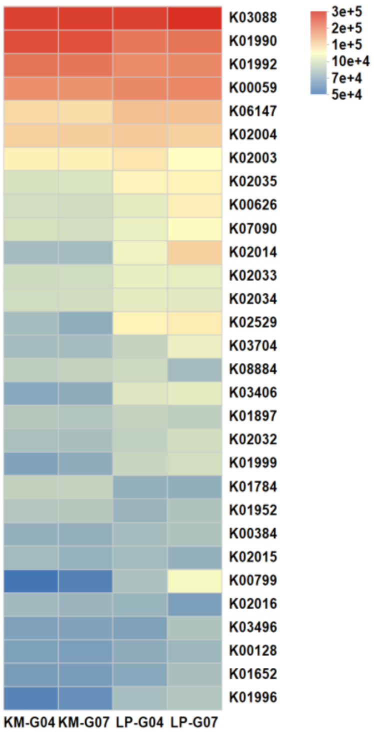

Figure S1. Heatmap of bacterial community functional predictions based on the KEGG database. KM-G04, KM-G07, LP-G04, and LP-G07 represent rhizosphere soil samples of ginger cultivars G04 and G07 grown at two planting locations, Kunming and Luoping, respectively.
